# Application of abdominal ultrasonography in surgical necrotizing enterocolitis: a retrospective study

**DOI:** 10.3389/fmicb.2023.1211846

**Published:** 2023-06-06

**Authors:** Qin Chen, Weiquan Yao, Fengdan Xu, Jinfeng Liao, Jinfeng Li, Minling Mai, Haoqiang Xie, Xiaoguang He, Ning Li

**Affiliations:** ^1^Department of Neonatal Intensive Care Unit, Dongguan Children's Hospital Affiliated to Guangdong Medical University, Dongguan, China; ^2^Department of Medical Imaging, Dongguan Children's Hospital Affiliated to Guangdong Medical University, Dongguan, China

**Keywords:** abdominal ultrasound, necrotizing enterocolitis, surgical, perforation, newborn infant

## Abstract

**Background:**

Necrotizing enterocolitis (NEC) is a severe inflammatory bowel disease that may lead to perforation, causing high morbidity and mortality in preterm infants. Abdominal ultrasound (AUS) has been shown to provide benefits in diagnosing and managing NEC in recent years.

**Objective:**

This study focused on the utility of AUS in the diagnosis and evaluation of surgical NEC.

**Patients and methods:**

In this retrospective study, available data of the patients diagnosed from January 2019 to June 2022 were reviewed. The sensitivity and specificity of AUS in diagnosing a perforation were analyzed. Typical cases for the application of AUS in monitoring and evaluating the progression, complications, and sequela of NEC were described.

**Results:**

There were 69 neonates diagnosed with NEC and examined by AUS, of whom eight patients developed a perforation. AUS was used for diagnosing a perforation in eight patients with key features of pneumoperitoneum and/or complex ascites, allowing us to find four locations of perforation, with a sensitivity and specificity of 100%.

**Conclusion:**

AUS plays an important role in diagnosing and evaluating surgical NEC in newborn infants, with good sensitivity and specificity.

## 1. Introduction

Necrotizing enterocolitis (NEC) is a devastating intestinal disease of preterm infants that can be difficult to diagnose and is characterized by overwhelming inflammation of the bowel wall, causing intestinal injury and necrosis (Epelman et al., 2007; Neu and Walker, 2011; Patel et al., 2015). Timely diagnosis and treatment are essential for better outcomes because NEC can rapidly progress to intestinal perforation, peritonitis, widespread inflammatory response syndrome, multiorgan dysfunction, and death (Cuna et al., 2022).

Diagnosis of NEC is made based on clinical presentation, laboratory tests, and imaging (Cuna et al., 2018). The modified Bell's classification remains the most important and widely used staging tool for neonates with NEC. Although abdominal radiographs (ARs) remain the imaging standard for evaluating NEC, an AR may miss up to 50% of the early signs of NEC. Pathognomonic findings, such as portal venous gas, pneumatosis, and pneumoperitoneum, can be difficult to identify the AR, and the absence of such findings cannot entirely exclude the diagnosis (Tam et al., 2002; Chan et al., 2021). Abdominal ultrasound (AUS) is a real-time feedback, non-invasive imaging modality. Various studies have demonstrated that AUS outperformed AR for diagnosis, management, and prediction of outcomes for NEC (Faingold et al., 2005; Epelman et al., 2007; Silva et al., 2007; Muchantef et al., 2013; Chen et al., 2018; van Druten et al., 2019; Alexander et al., 2021). AUS can well display the signs of bowel wall echo, peristalsis, blood perfusion of the bowel wall, pneumatosis intestinalis, ascites, and free gas with sensitivity and specificity of up to 70 and 80%, respectively. The sensitivity of AUS in intestinal necrosis (76.9 vs. 38.5%) and intestinal perforation (61.5 vs. 15.4%) is higher than that of X-ray (Tracy et al., 2020). In a recent study of a pilot diagnostic randomized clinical trial, the rate of disagreement between paired AR and AUS can be 25%, with all cases of disagreement arising from pneumatosis intestinalis (Cuna et al., 2022). When used in conjunction with color Doppler, bowel wall perfusion can be evaluated (Faingold et al., 2005; Epelman et al., 2007; Hwang et al., 2022). AUS may be a useful adjunct in detecting changes consistent with NEC even when radiographs are inconclusive. However, the adoption of robust AUS programs into clinical practice has been slow (Ben Fadel et al., 2019; Miller et al., 2020).

This article focuses on the emerging role of AUS in the early diagnosis of surgical NEC, the benefits of AUS in daily practice, and the evaluation of complications and sequela through illustrative cases. We found that AUS had very good sensitivity and specificity for the diagnosis of perforation based on factors such as pneumoperitoneum, complex ascites, and intestinal wall blood supply. Focal fluid collections or echogenic fluid were predictors of the eventual need for surgery and served as evidence of perforation, even if pneumoperitoneum is not seen. Therefore, the significance of complex ascites in the diagnosis of NEC perforation should be emphasized.

## 2. Patients and methods

### 2.1. Patients

This retrospective study was approved by the Research Ethics Committee of Dongguan Children's Hospital (no. LL2022112901). Available data of patients with NEC admitted urgently to the neonatal intensive care unit (NICU) and patients diagnosed with NEC during hospital stay from January 2019 to June 2022 were reviewed. The sensitivity and specificity of AUS in diagnosing a perforation were analyzed. Typical cases for the application of AUS in monitoring and evaluating the progression, complications, and sequela of NEC were described. The inclusion criteria were as follows: (1) meeting the criteria of Modified Bell's NEC stage of III; (2) gestational age at birth ≤ 44 weeks; (3) postnatal age ≤ 28 days for term neonates and postmenstrual age ≤ 44 weeks for preterm neonates at NEC onset; and (4) bedside AUS performed frequently from the early onset of NEC or urgent admission for NEC diagnosis and prognosis evaluation. The exclusion criteria were as follows: (1) intestinal malformations and (2) severe persistent pulmonary hypertension.

### 2.2. Abdominal ultrasonography

#### 2.2.1. Equipment

A portable CXR (MUX-10J) was used in this study. Abdominal computed tomography (CT) was acquired with a 16-slice spiral CT scanning unit (Brilliance, Philips Co.). A portable ultrasound machine (CX50, Philips Co.) was used regularly in our department, and a linear array probe with a frequency of 9–13 MHz was used in all AUS scans.

#### 2.2.2. AUS and AR examination method

Every AUS examination was performed and read by a pediatric ultrasound expert, while AR was performed and read by pediatric radiologists. The AUS expert and AR radiologists were separate physicians. Each baby was placed in the supine position, and each quadrant of the abdomen was systematically assessed by scanning in transverse and sagittal planes. CD AUS at a velocity of 0.029–0.11 m/s evaluated intestinal mural blood flow (Faingold, 2018). The abdomen was imaged anteriorly and posteriorly by AR, and abdominal upright and horizontal projection images were documented.

#### 2.2.3. Observation indexes

Key AUS features of NEC were observed carefully (Tam et al., 2002; Faingold et al., 2005; Epelman et al., 2007; Silva et al., 2007; Muchantef et al., 2013; Patel et al., 2015; Allin et al., 2017; Erinumberger, 2017; Chen et al., 2018; Faingold, 2018; Markel and Underwood, 2018; Ben Fadel et al., 2019; van Druten et al., 2019; Miller et al., 2020; Tracy et al., 2020; Alexander et al., 2021; Chan et al., 2021; Gao et al., 2021; Kotb et al., 2021; Cuna et al., 2022; Hwang et al., 2022). These features included (1) bowel wall thickness: normal between 1 and 2–2.7 mm; (2) decreased intestinal peristalsis: intestinal peristalsis <10 times/min. (3) Pneumatosis intestinalis (PI): granular, stripe, ring-shaped hyperechoic foci in the intestinal wall; (4) portal venous gas (PVG): movable granular hyperechoic foci within the portal vein and its branches; (5) pneumoperitoneum: free intraperitoneal air seen as echogenic foci or stacked echogenic lines outside the bowel lumen; (6) ascites: detectable free liquid dark areas in the abdominal cavity (simple free fluid was seen as anechoic regions surrounding intraperitoneal structures, while complex free fluid was seen as echogenic, with possible loculations); and (7) bowel perfusion: increased perfusion with >9 dots of color Doppler signal per square centimeter (Alexander et al., 2021; Gao et al., 2021). The key AR features of NEC included PVG, pneumoperitoneum, bowel dilatation, and reduced inflation (van Druten et al., 2019).

Diagnosis of NEC can be performed when pathognomonic signs, such as PVG or PI, are present. The indication for a surgical procedure in NEC is the presence of perforation, which manifests as pneumoperitoneum and/or complex ascites (van Druten et al., 2019; Alexander et al., 2021). The correlation between the ASU feature and modified Bell's Stage Criteria referred to the study by van Druten et al. (2019).

#### 2.2.4. Statistical analysis

The statistical analysis was performed using SPSS software (v. 19.0). Descriptive statistical methods were used to describe the study population. Numerical data were expressed as the mean ± standard deviation (SD). Categorical variables were presented as frequencies or rates. A two-sided *p*-value of <0.05 was considered significant.

## 3. Results

### 3.1. Clinical manifestations of NEC

**Figure 1:**
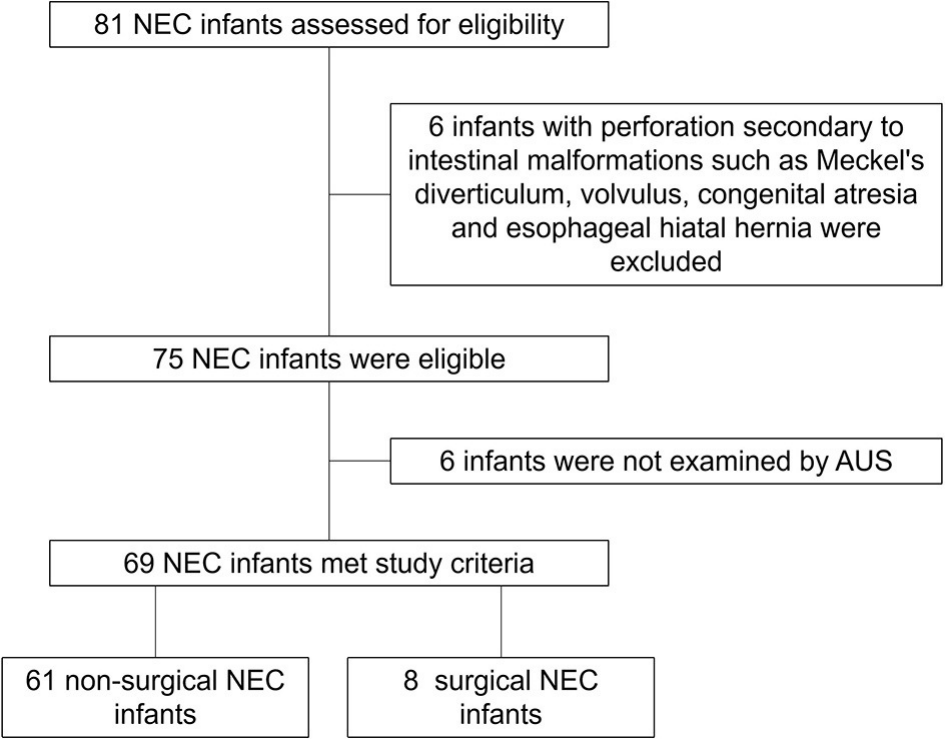
Patient disposition.

**Figure 2:**
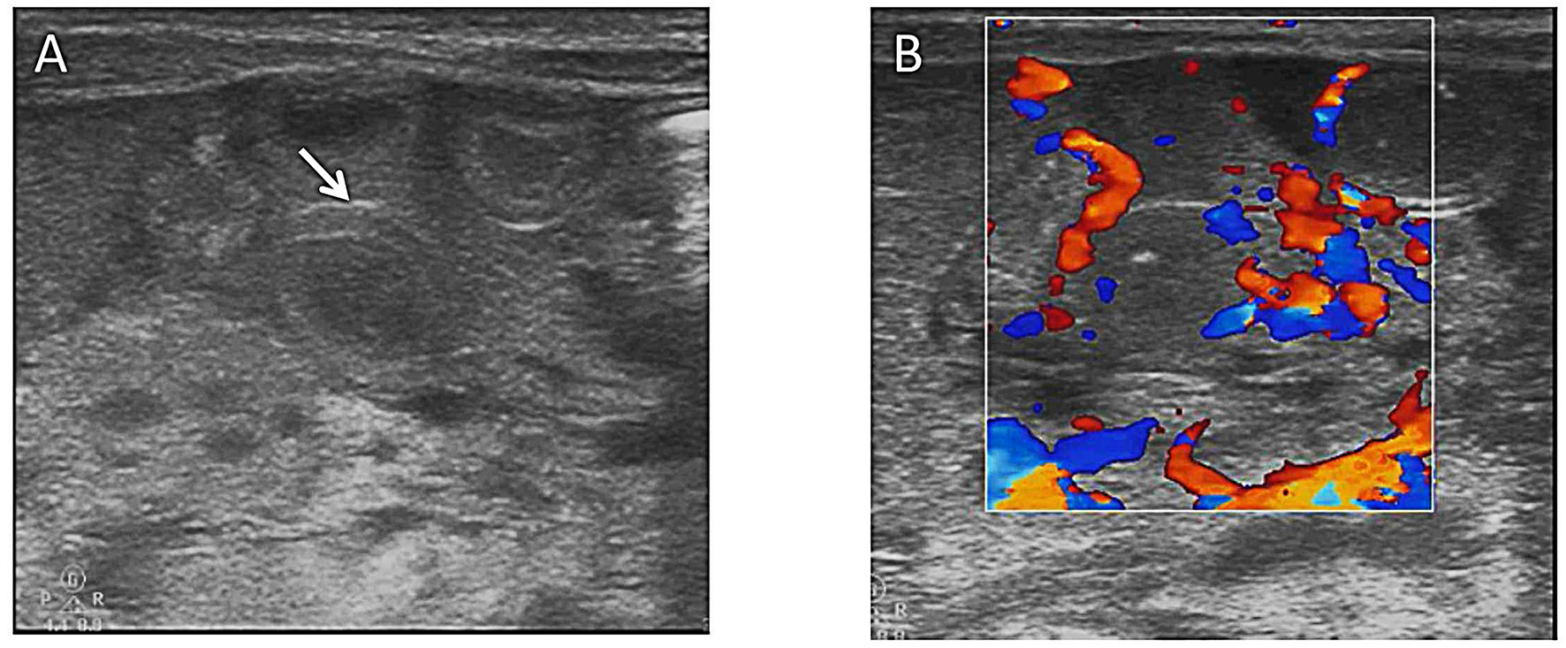
**(A)** Bowel wall thickening (arrows) on abdominal ultrasound. **(B)** Colored ultrasound revealed increased bowel wall perfusion.

**Figure 3:**
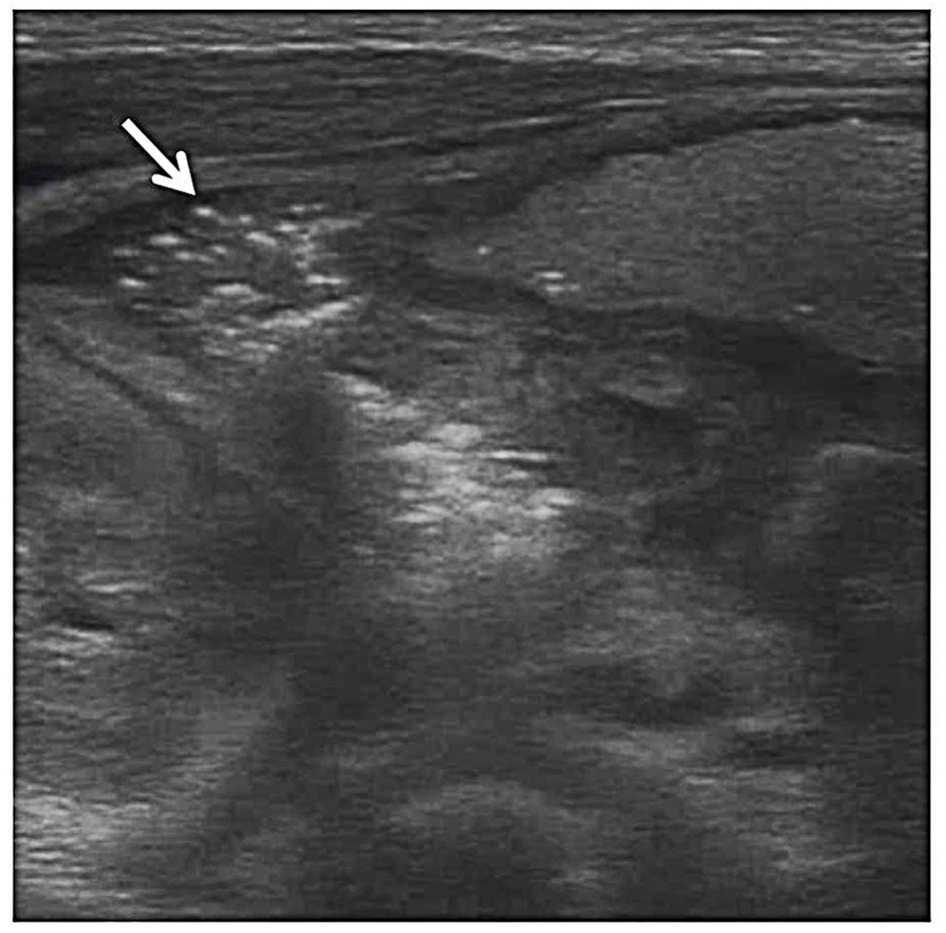
Pneumatosis intestinalis. Grayscale ultrasound shows echogenic foci (arrow) within the bowel wall indicative of gas bubbles within the bowel wall.

**Figure 4:**
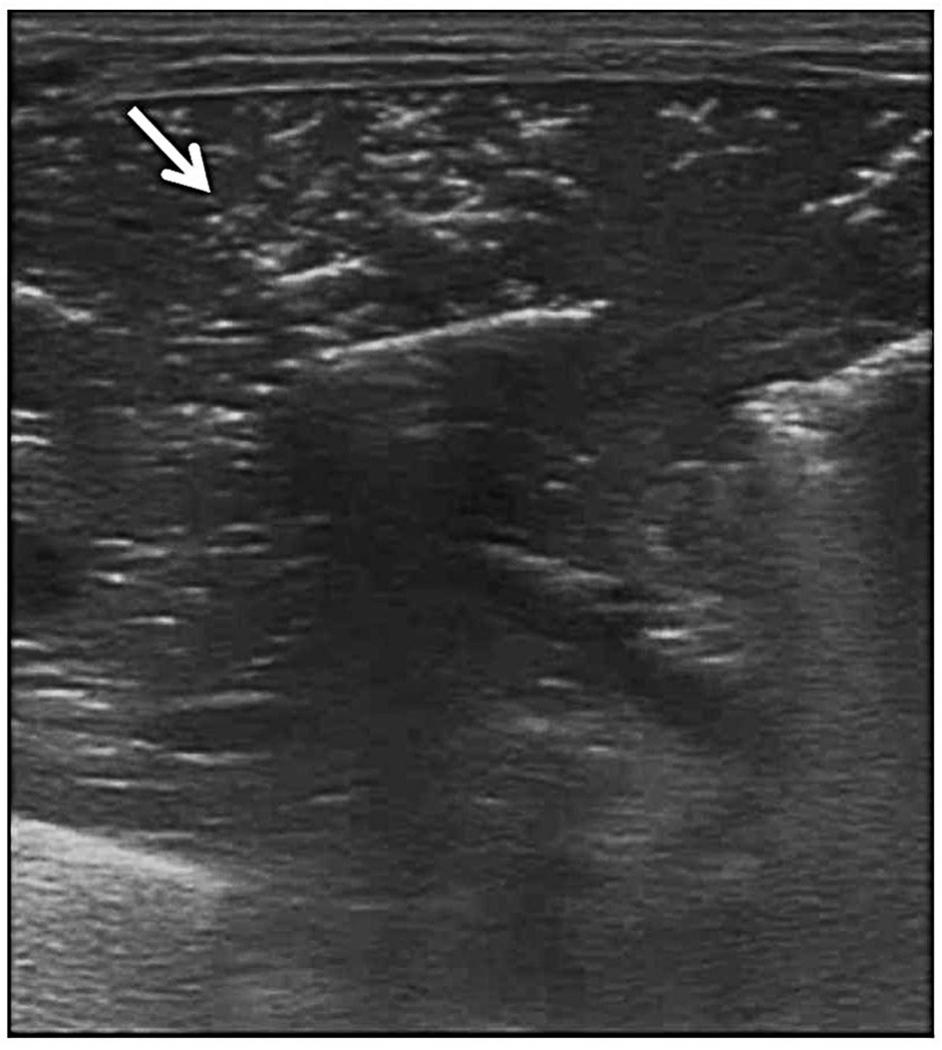
Portal venous gas. Grayscale ultrasound shows portal venous gas from the small intestine (arrow).

There were 81 neonates diagnosed with NEC (we excluded six patients diagnosed with perforation secondary to intestinal malformations, such as Meckel's diverticulum, volvulus, congenital atresia, and esophageal hiatal hernia, and six patients who were not examined by AUS) in our NICU from January 2019 to June 2022, and 69 of them were examined by AUS (Figure 1). Among these 69 patients, 11 met the criteria of Bell stage II, 10 met the criteria of Bell stage III, and perforation occurred in eight patients (Table 1) who underwent surgery and survived. AUS was used for the diagnosis of perforation in eight patients (Table 2). Figures 2–4 showed key AUS features of NEC of stage level I to IV.

**Table 1:** General characteristics of patients with NEC.

**Characteristic**	**Data**
Male [*n* (%)]	35 (50.7)
GA [(mean ± SD), week]	33.6 ± 3.7
BW [(mean ± SD), g]	1,961 ± 728
Cesarean delivery [*n* (%)]	38 (55.1)
**Bell's stage of NEC**	
IIIB [*n* (%)]	8 (12.9)
IIIA [*n* (%)]	2 (2.9)
IIB [*n* (%)]	2 (2.9)
IIA [*n* (%)]	9 (12.9)
I [*n* (%)]	48 (68.6)

**Table 2:** Clinical manifestations of patients with surgical NEC.

**Case**	**1**	**2**	**3**	**4**	**5**	**6**	**7**	**8**
**Sex**	**Female**	**Male**	**Male**	**Female**	**Female**	**Male**	**Male**	**Female**
GA (weeks)	34^+2^	29^+6^	39	31^+2^	33	39^+1^	32^+2^	32^+1^
Mode of delivery	Vaginal	Cesarean section	Vaginal	Cesarean section	Cesarean section	Vaginal	Cesarean section	Cesarean section
BW (g)	2,220	1,460	3,350	1,215	1,500	2,690	2,145	1,215
Age of onset of perforation diagnosed (days)	6	2	2	6	14	5	12	12
Abd. distension	Yes	Yes	Yes	Yes	Yes	No	Yes	Yes
Gastric residue	No	No	No	No	Yes	Yes	No	No
Bloody stools	Yes	No	No	No	No	No	No	No
Abd. mass	No	No	No	No	Yes	No	No	No
WBC (× 10^9^/L)	8	15.3	2.98	18.4	20.96	0.99	2.03	8.9
NEU (%)	78.6	62.3	23.9	72.6	61.2	55.6	65.5	74.5
PLT (× 10^9^/L)	156	203	107	208	146	130	527	196
CRP (mg/L)	8.1	5	11.7	24.87	3.44	159.99	20.4	191.96
PCT (ng/ml)	36.1	3.46	68.29	20.05	0.17	5.52	< 0.25	46.51
Outcomes	Survived	Survived	Survived	Survived	Survived	Survived	Survived	Survived

GA, gestational age; BW, body weight; WBC, white blood cell; NEU, neutrophil; PLT, platelet; CRP, C-reactive protein; PCT, procalcitonin.

### 3.2. Sensitivity and specificity of AUS in diagnosing surgical NEC

**Figure 5:**
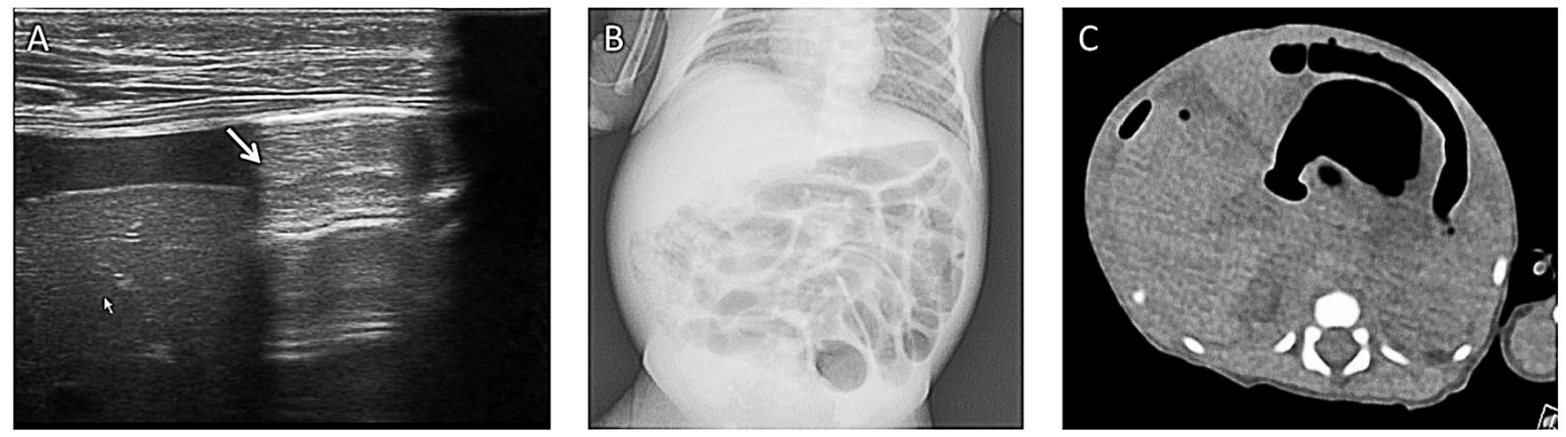
Pneumoperitoneum. A boy (gestational age of 32 weeks) developed abdominal distension and bloody stool on his 14th day. **(A)** Grayscale ultrasound shows an echogenic interface just below the abdominal wall (arrow) with dirty posterior acoustic shadowing that is from free air in the abdomen. **(B)** This air was not visible on the corresponding supine abdominal radiograph. **(C)** The following CT examination supported the result of AUS. This case demonstrates that ultrasound is more sensitive to pneumoperitoneum.

**Figure 6:**
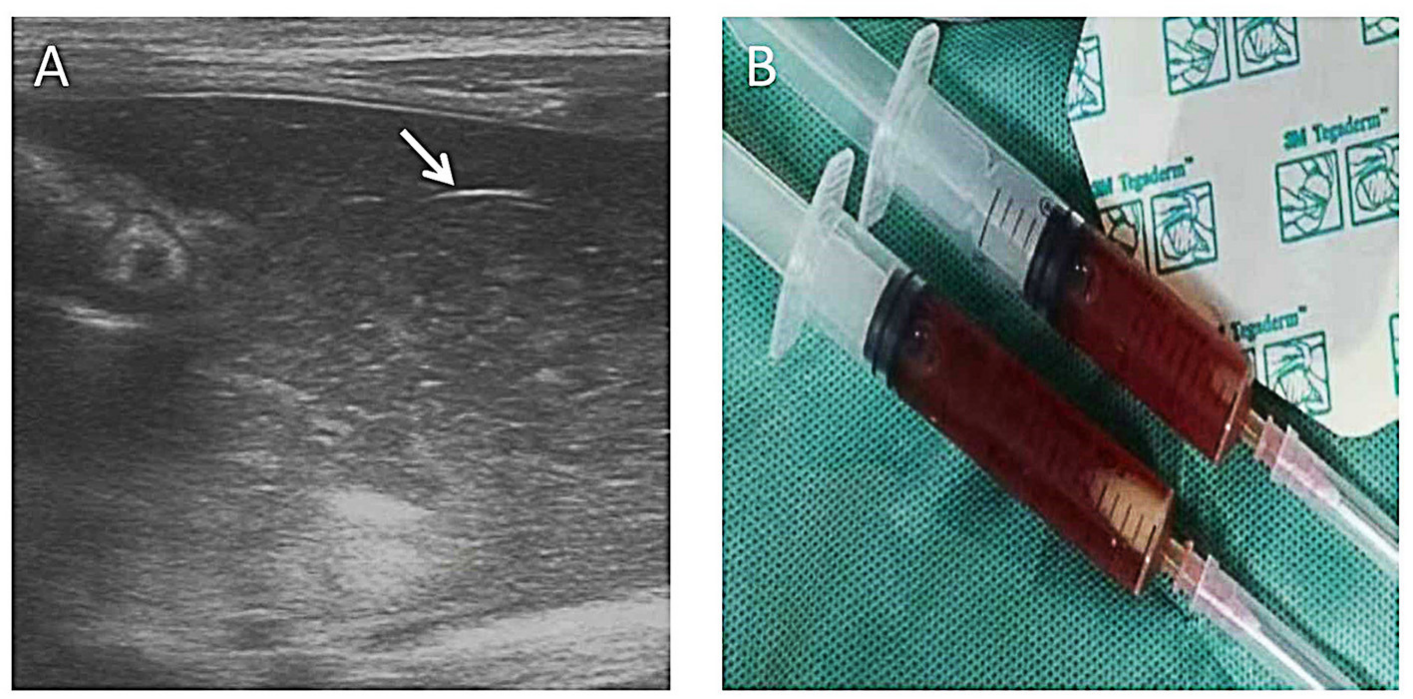
Complex ascites. A 10-day-old girl (gestational age of 32 weeks), born by cesarean section due to the mother's placental abruption, developed abdominal distension and bloody stools and rapidly progressed to peritonitis and septic shock. A mass could be felt in the right lower abdomen. AR showed a lot of free fluid. AUS showed complex ascites, bowel wall thinning, decreased intestinal peristalsis, and decreased blood perfusion in the bowel wall. Neither perforation nor pneumoperitoneum was found by AUS. The diagnostic ascites puncture showed thick bloody ascites. Bowel wall perforation was considered and proven during the surgery. **(A)** Layering debris within the collection of free fluid (long arrow) and adjacent loop of the thickened bowel wall (short arrow). **(B)** Ascites aspiration.

Among the eight patients who underwent surgery, AUS was used for the diagnosis of perforation. In these patients, both the sensitivity and specificity of AUS in diagnosing a perforation due to NEC were 100%. There were seven images of pneumoperitoneum and seven images of complex ascites. Decreased bowel wall perfusion was seen in two cases, and locations of perfusions (two in the small intestine and two in the colon) were found in four patients (Table 3). Figure 5 showed Pneumoperitoneum and Figure 6 showed Complex ascites.

**Table 3:** Clinical manifestations of patients with surgical NEC.

**Case**	**1**	**2**	**3**	**4**	**5**	**6**	**7**	**8**
**Pneumoperitoneum**	**Yes**	**Yes**	**Yes**	**Yes**	**Yes**	**No**	**Yes**	**Yes**
Location of perforation	Yes, on intestinal	Yes, on the colon	No	Yes, on intestinal	No	No	No	Yes, on the colon
Complex ascites	Yes	Yes	Yes	Yes	No	Yes	Yes	Yes
Decreased bowel perfusion	No	No	No	No	Yes	Yes	No	No

### 3.3. Typical cases for AUS in evaluating complications and sequela of NEC

#### 3.3.1. Identifying the location of a perforation

**Figure 7:**
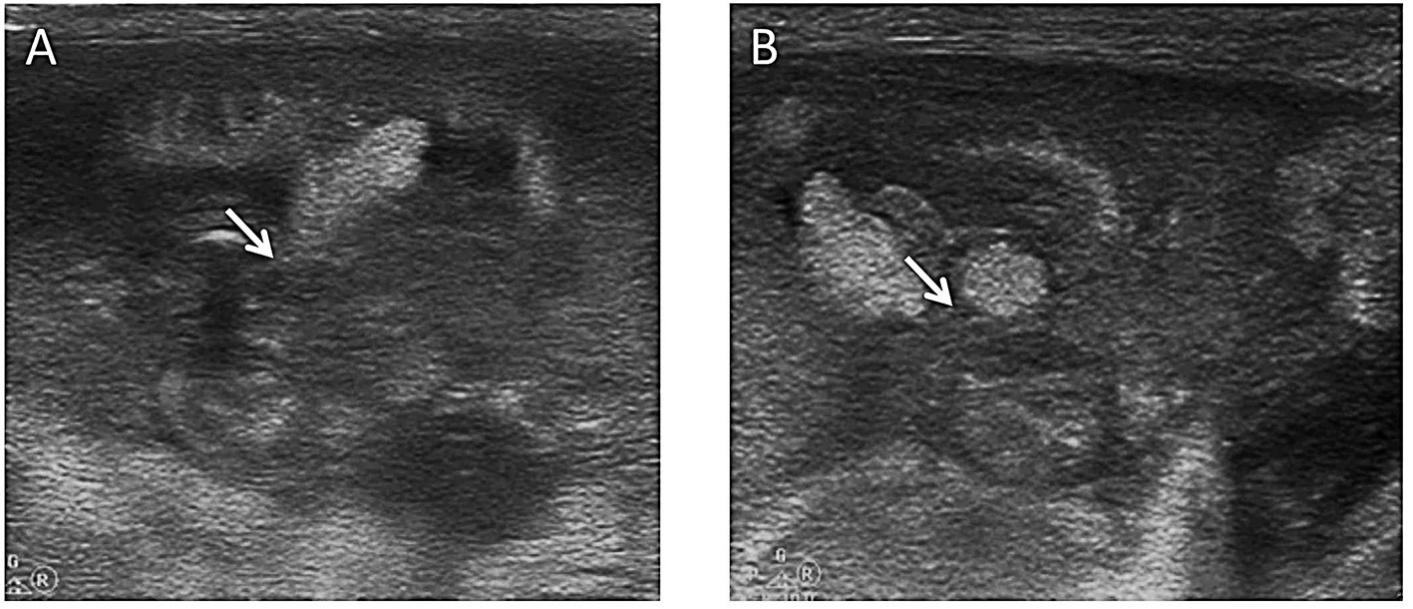
Location of perforation. An extra low birth weight boy (28 weeks of gestation) presented with abdominal distension on the 2nd day of life. Although medical therapy including fasting, antibiotics, and mechanical ventilation was given, the baby's condition kept worsening, and pneumoperitoneum was proven by an abdominal CT scan. He was transferred to our NICU for surgery. **(A)** Ultrasound showed that the continuity of the mid-abdominal small bowel wall was interrupted, and a hyperechoic mass (a fecal stone) was found at the perforation (arrow). **(B)** This image showed that the contents were flowing from the perforation in the bowel wall (arrow) ~10 min later.

The universally accepted indication for a surgical procedure in NEC is the presence of perforation (Cuna et al., 2018; van Druten et al., 2019). The prognosis for patients with NEC worsens after bowel perforation. Pneumoperitoneum, bowel necrosis, complex abdominal fluid, and intestinal obstruction on imaging are common indications for surgery in neonates with NEC. Perforation also may lead to an accumulation of complex peritoneal fluid alone without free air (Cuna et al., 2018; van Druten et al., 2019) (Figure 7).

#### 3.3.2. Monitoring the progression of patients with deteriorating NEC

Rapid progression of NEC requires frequent monitoring by AUS and reduced X-ray exposure. Portal venous gas, pneumatosis intestinalis, bowel wall thickening, intestinal motility, and peritoneal dropsy should be observed carefully at the bedside. Evaluation of the bowel wall perfusion using the color Doppler helps assess bowel viability and temporal evolution of NEC when compared to other structures (Alexander et al., 2021).

*Case A*. A girl patient aged 26 + 3 weeks (gestational age), the eldest of twins, was born vaginally. She received donated breastfeeding. On the 43rd day, she and her twin brother developed abdominal distension, blood stools, and then septic shock. White blood cell (WBC) count from the peripheral blood decreased, while C-reactive protein (CRP) and procalcitonin (PCT) increased significantly. AUS showed intestinal wall thickening, decreased peristalsis, and massive simple ascites, indicating stage NEC IIIA. As the baby's condition rapidly deteriorated, AUS was used to assess the development of NEC in the acute stage twice a day. On the 1st day of the onset of NEC, AUS showed thickened intestinal wall, pneumatosis, and decreased peristalsis, while bowel wall perfusion and simple ascites, the deepest of which was ~10 mm, increased. On the 2nd day, AUS showed diffuse bowel wall edema, more pneumatosis, and decreased intestinal peristalsis. However, rich blood flow signals still could be detected in the bowel wall, and the deepest free fluid decreased to 8 mm. Medical treatment was continued because AUS did not show signs of worsening NEC, such as intestinal wall thinning, the disappearance of intestinal peristalsis, and decrease in intestinal wall blood supply, as well as surgical indicators, such as complex ascites, perforation, and pneumoperitoneum. On the 3rd day, AUS showed a reduction in bowel edema and pneumatosis, indicating improvement in the condition. On the 10th day, AUS showed that intestinal edema significantly subsided and intestinal motility returned to normal. Thus, gastrointestinal feeding was started. On the 14th day, AUS showed that intestinal lesions disappeared.

#### 3.3.3. NEC complicated with intussusception

**Figure 8:**
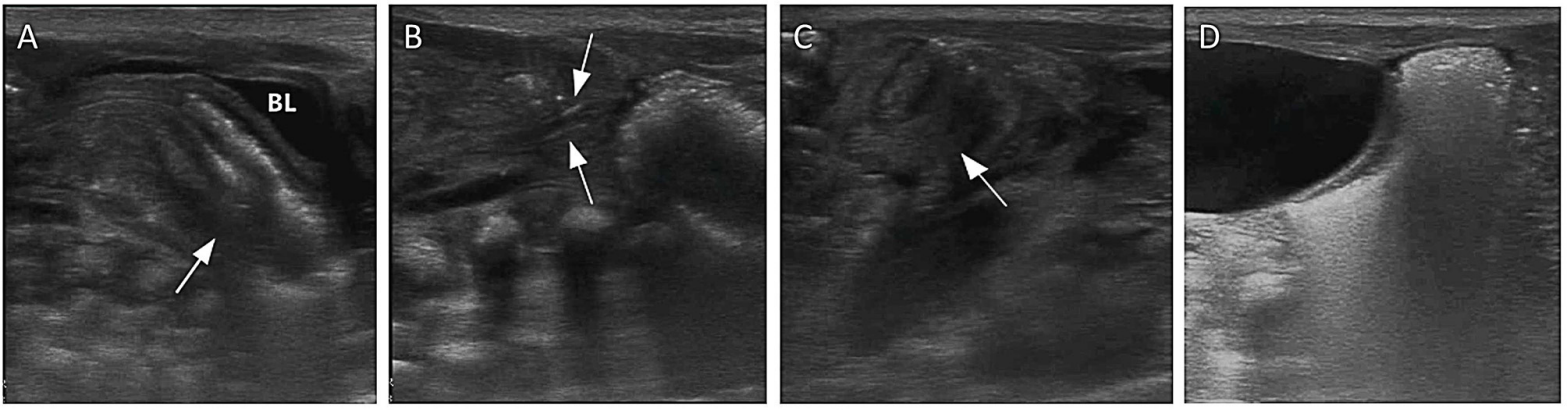
Images show NEC complicated by intussusception as monitored by AUS. **(A)** A mass with a “concentric circle sign” and “sleeve sign” was seen in the rectum, and the sigmoid colon was considered to be inserted into the rectum (arrow). **(B)** Gas was seen flowing directly into the mesenteric vein from the location of intussusceptions (arrow). **(C)** The intussusception mass in the rectum gradually retreated into the sigmoid colon within minutes (arrow). **(D)** The sigmoid colon exited from the rectum after ~3 h, and stool was well filled in the sigmoid colon.

*Case B*. The younger brother of the above-described twins, sharing the same donated breastfeeding with his older sister, developed abdominal distension, bloody stools, and septic shock at the age of 43 days and was diagnosed with NEC on the same day. On the 1st day of the NEC onset, a mass echo with a concentric circle sign and false kidney sign in the rectum was detected by AUS, indicating sigmoid-rectal intussusception. AR image performed 30 min before showed the signs of NEC and no bowel obstruction or intussusceptions. Approximately 3 h later, AUS showed that the mass disappeared, the sigmoid tube wall was thickened, the echo was reduced, and several indentation echoes appeared, indicating sigmoid colitis and a small ulcer. During the whole observation, abundant blood perfusion was visible in the intestinal wall. On the 3rd day, the gas in the portal vein disappeared, the intestinal tube inflated, and the peristalsis improved. No intussusception or intestinal obstruction was found on the following AUS examinations. The boy recovered after a 2-week treatment (Figure 8).

#### 3.3.4. Sequential evaluation of a post-NEC intestinal stricture

**Figure 9:**
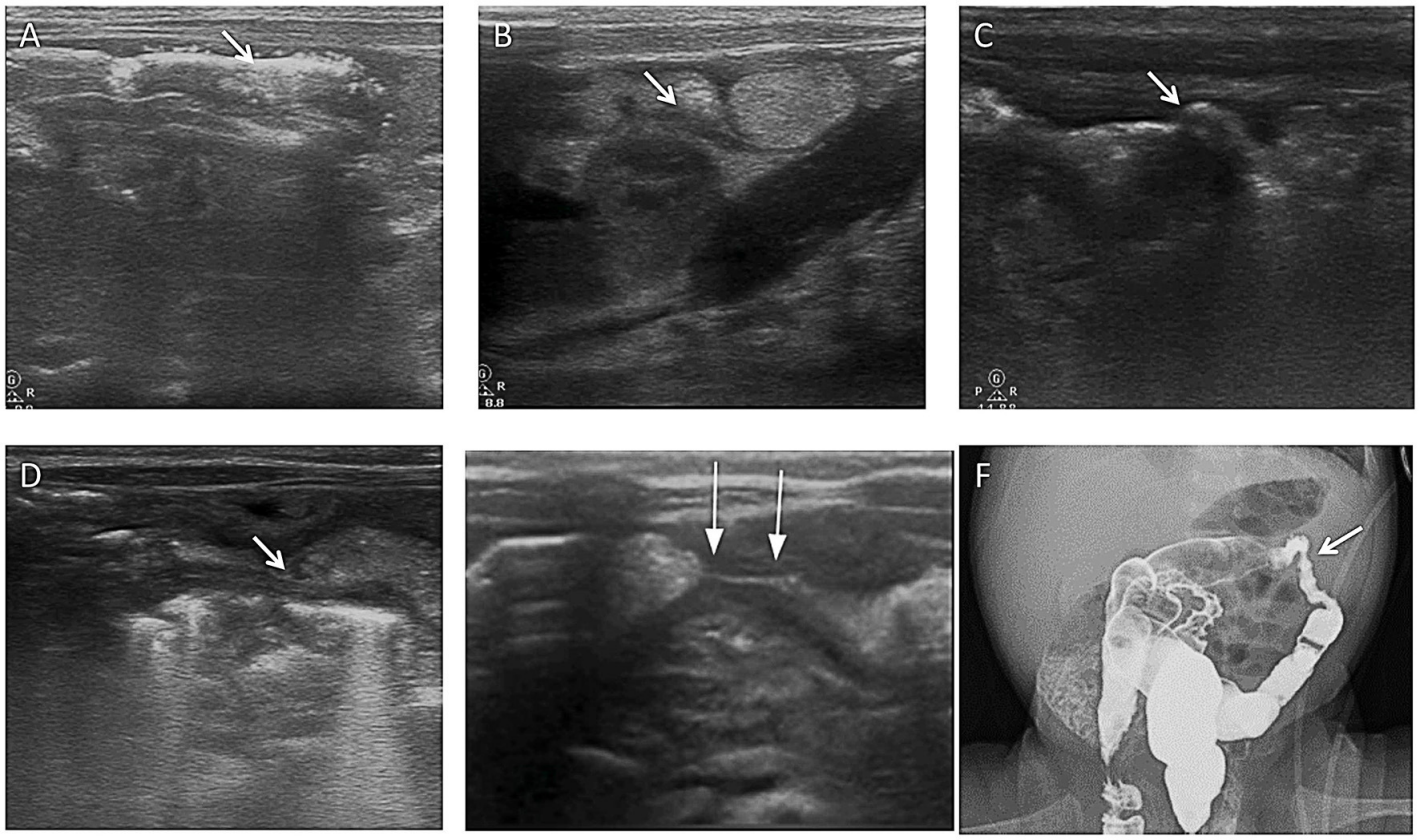
Images show a post-NEC intestinal stricture evaluated by AUS. **(A)** Multiple mural gases were seen in the transverse colon on the 1st day of NEC (arrow). **(B)** After 11 days, the wall of the transverse colon was irregularly thickened (arrow). **(C)** On the 24th day, the wall of the transverse colon was irregularly thickened, and the intestinal lumen was stiff and irregular (arrow). **(D)** On the 38th day, the wall of the transverse colon was irregularly thickened, the shape of the intestinal lumen was rigid, and the intestinal lumen was narrow (arrow). **(E)** On the 61st day, the wall of the transverse colon was irregularly thickened, the shape of the intestinal lumen was rigid, and significant stenosis of the intestinal lumen was seen (arrow). **(F)** On the 61st day, total gastrointestinal radiography with Iohexol showed severe stenosis in the colon, proving the result by AUS (arrow).

*Case C*. A 32-week-old premature infant presented with bloody stool and abdominal distension on the 7th day of life and was diagnosed with NEC stage IIB, followed by discharge after medical treatment. The patient was followed up with AUS for NEC sequelae. Intestinal wall stiffness appeared on the 30th day of NEC onset and worsened gradually on ASU every 2 weeks. In the 3rd month after the NEC onset, the baby developed obvious abdominal distension and difficult defecation. AUS showed severe intestinal stenosis, and the subsequent AR angiography results were consistent. Therefore, the patient underwent surgical treatment (Figure 9).

## 4. Discussion

Necrotizing enterocolitis is one of the leading causes of morbidity and mortality in preterm neonates and particularly affects low birth weight (LBW) infants in NICUs. While NEC may progress rapidly and the mortality rate is higher after perforation, the transition from medical to surgical care represents a critical decision-making point in NEC management that can have important implications for clinical outcomes (Cuna et al., 2018). The International Neonatal Consortium's NEC subgroup was convened in 2017 to revisit the diagnostic and classification criteria for NEC (Erinumberger, 2017; Markel and Underwood, 2018). In 2020, the Point-of-Care Ultrasound (POCUS) Working Group of the European Society of Pediatric and Neonatal Intensive Care (ESPNIC) recommended appropriate indications for AUS for NEC: (1) establishing an early diagnosis of NEC; (2) establishing the diagnosis of NEC when AR is equivocal; (3) AR demonstrating a gasless abdomen; (4) evaluating for complications in known NEC; and (5) evaluation of features suggestive of the need for surgical intervention in the setting of clinical deterioration (Singh et al., 2020).

The mortality rate is higher after perforation; thus, earlier detection of severely ischemic or necrotic bowel loops, before perforation occurs, could improve the morbidity and mortality in NEC (Allin et al., 2017). A meta-analysis revealed that bowel wall thickening [odds ratio (OR) 8.58, 95% confidence interval (CI) 3.42–21.53], absent peristalsis (OR: 10.68, 95% CI 1.65–69.02), absent perfusion (OR: 6.99, 95% CI 2.06–23.76), and complex ascites (OR: 11.28, 95% CI 4.23–30.04) were associated with an increased risk of surgery or death, while an increase in bowel perfusion was not associated with surgery or death in NEC (OR: 2.60, 95% CI 0.61–11.13) (Cuna et al., 2018). Lazow et al. (2021) suggested four predictors of surgical NEC: abdominal wall erythema (OR: 8.2, *p* = 0.048), portal venous gas on AXR (OR: 29.8, *p* = 0.014), echogenic free fluid (OR: 17.2, *p* = 0.027), and bowel wall thickening (OR: 12.5, *p* = 0.030) on AUS. Another study has shown that in stage IIIa, 71% of cases had the absence of bowel peristalsis and intramural gas, and the absence of perfusion and bowel wall thinning of <1 mm was found in 86% of neonates (Aliev et al., 2017). Bowel wall thickness, bowel wall texture, bowel perfusion, and bowel peristalsis are all much more difficult to evaluate by AR compared with AUS. Changes in these AUS variables can provide earlier evidence of disease progression before bowel perforation. Additionally, every 6 h, AR monitoring for NEC exposes vulnerable neonates to ionizing radiation, which might increase lifetime cancer mortality risk up to 4.3- to 20-fold (Baird et al., 2013). Therefore, AUS is more advantageous in monitoring the progression of NEC compared with AR. AUS was used to partially replace AR as a routine assessment method for diagnosed NEC (Bell's stages II and III) in our department. As a result, the AR exposure was significantly reduced. As mentioned above, although the baby's condition (Case 1) was severe, there were no signs of NEC deterioration, such as the absence of bowel perfusion, peristalsis, and perforation indicators including pneumoperitoneum and complex ascites, suggesting that surgical intervention was not needed at that time.

Once perforation occurs, the earlier the diagnosis, the higher the survival rate. AUS has unique advantages in the detection of perforation compared with AR. First, sensitivity and specificity for pneumoperitoneum are higher. Second, AUS directly displays the location of the perforation. Third, AUS reveals complex ascites, indicating perforation. In eight NEC patients with bowel perforation detected by ultrasound in our NICU, AUS had a sensitivity of 100% and a specificity of 100% for the diagnosis of perforation based on factors such as pneumoperitoneum, complex ascites, and intestinal wall blood supply. In four patients, AUS also directly identified the perforation and its location. All the above results were confirmed by surgery. In eight cases, seven patients showed pneumoperitoneum with complicated ascites, and in the other case, only complicated ascites without pneumoperitoneum (neither pneumoperitoneum nor perforation was found on AR and abdominal CT) was detected. Perforation can also lead to an accumulation of complex peritoneal fluid alone without free air (Palleri et al., 2017; Cuna et al., 2018). Focal fluid collections or echogenic fluid are predictors of the eventual need for surgery and are evidence of perforation, even if pneumoperitoneum is not seen. In these cases, surgical consultation is warranted (van Druten et al., 2019). Therefore, the significance of complex ascites in the diagnosis of NEC perforation should be emphasized.

Although intussusception can rarely be detected in the neonatal period, triggers of NEC, such as prematurity, hypoxia, and low intestinal perfusion, may be a risk factor for intussusception due to stricture formation and intestinal dysmotility (Taskinlar et al., 2014). Due to the similar symptomatology of NEC and intussusception and the high prevalence of NEC in this age group, AUS is recommended for differential diagnosis to avoid the delayed treatment of intussusception in premature infants. A systematic review including 43 studies with a total of 52 cases of intussusception reported between 1963 and 2020 has revealed that the most common location was the small bowel, detecting colo-colic intussusception in two cases only (Kotb et al., 2021). A sigmoid-rectal intussusception (without perforation) was found in a preterm during monitoring the progression of stage IIIA NEC, which disappeared after a few hours. Intussusception was considered to be secondary to NEC based on the similar sepsis onset at the same time compared with his twin sister.

Post-NEC intestinal stricture is estimated to affect 15%−40% of NEC survivors and is associated with severe and prolonged morbidity, with 15%−30% of patients being treated medically and 20%−43% of patients being treated surgically (Phad et al., 2014; Burnand et al., 2016). The treatment of post-NEC strictures, particularly asymptomatic strictures, is controversial regarding the modalities of screening, indications for surgery, and optimal time. Sequential evaluation by AUS might help choose the therapeutic schedule and find indications for surgery and optimal time. We traced patients with NEC of stage level IIB (two cases) and level III (11 cases including nine patients who underwent surgery) by AUS, and only one post-NEC intestinal stricture was found. The development of intestinal strictures was documented and evaluated, helping to make a decision for surgeons.

## 5. Conclusion

Abdominal ultrasound is a real-time feedback, non-invasive, radiation-free option ensuring a high degree of safety, portability, and accessibility. AUS has great advantages in enhancing the detection of NEC, determining management, and predicting outcomes. Furthermore, AUS had very good sensitivity and specificity for the diagnosis of perforation based on factors such as pneumoperitoneum, complex ascites, and intestinal wall blood supply. Focal fluid collections or echogenic fluid are predictors of the eventual need for surgery and serve as evidence of perforation, even if pneumoperitoneum is not seen. Therefore, the significance of complex ascites in the diagnosis of NEC perforation should be emphasized. It is thus recommended for medical staff in NICUs to promote AUS and become proficient in performing AUS.

## Data availability statement

The original contributions presented in the study are included in the article/supplementary material, further inquiries can be directed to the corresponding authors.

## Ethics statement

The studies involving human participants were reviewed and approved by Dongguan Chlidren's Hospital. Written informed consent to participate in this study was provided by the participants' legal guardian/next of kin. Written informed consent was obtained from the minor(s)' legal guardian/next of kin for the publication of any potentially identifiable images or data included in this article.

## Author contributions

QC, WY, FX, JLia, and JLi performed the experiments. MM and HX conducted statistical data analysis. QC and NL wrote the manuscript. XH and NL designed the study and revised the manuscript. All authors read and approved the final manuscript.

## References

[B1] Alexander K. M. Chan S. S. Opfer E. Cuna A. Fraser J. D. Sharif S.. (2021). Implementation of bowel ultrasound practice for the diagnosis and management of necrotizing enterocolitis. Arch. Dis. Child Fetal Neonatal Ed. 106, 96–103. 10.1136/archdischild-2019-318382 32398270 PMC7788207

[B2] Aliev M. M. Dekhqonboev A. A. Yuldashev R. Z. (2017). Advantages of abdominal ultrasound in the management of infants with necrotizing enterocolitis. Pediatr. Surg. Int. 33, 213–216. 10.1007/s00383-016-4017-8 27822782

[B3] Allin B. Long A. M. Gupta A. Knight M. Lakhoo K. British Association of Paediatric Surgeons Congenital Anomalies Surveillance System Necrotising Enterocolitis Collaboration (2017). A UK wide cohort study describing management and outcomes for infants with surgical necrotising enterocolitis. Sci. Rep. 7, 41149. 10.1038/srep41149 28128283 PMC5269581

[B4] Baird R. Tessier R. Guilbault M.-P. Puligandla P. Saint-Martin C. (2013). Imaging, radiation exposure, and attributable cancer risk for neonates with necrotizing enterocolitis. J. Pediatr. Surg. 48, 1000–1005. 10.1016/j.jpedsurg.2013.02.016 23701773

[B5] Ben Fadel N. Pulgar L. Khurshid F. (2019). Point of care ultrasound (POCUS) in Canadian neonatal intensive care units (NICUs): where are we? J. Ultrasound 22, 201–206. 10.1007/s40477-019-00383-4 31073871 PMC6531515

[B6] Burnand K. M. Zaparackaite I. Lahiri R. P. Parsons G. Farrugia M. K. Clarke S. A.. (2016). The value of contrast studies in the evaluation of bowel strictures after necrotising enterocolitis. Pediatr. Surg. Int. 32, 465–470. 10.1007/s00383-016-3880-7 26915085

[B7] Chan B. Gordon S. Yang M. Weekes J. Dance L. (2021). Abdominal ultrasound assists the diagnosis and management of necrotizing enterocolitis. Adv. Neonatal Care 221, 365–370. 10.1097/ANC.0000000000000919 34469367

[B8] Chen S. Hu Y. Liu Q. Li X. Wang H. Wang K. (2018). Comparison of abdominal radiographs and sonography in prognostic prediction of infants with necrotizing enterocolitis. Pediatr. Surg. Int. 34, 535–541. 10.1007/s00383-018-4256-y 29602968

[B9] Cuna A. Chan S. Jones J. Sien M. Robinson A. Rao K.. (2022). Feasibility and acceptability of a diagnostic randomized clinical trial of bowel ultrasound in infants with suspected necrotizing enterocolitis. Eur. J. Pediatr. 181, 3211–3215. 10.1007/s00431-022-04526-4 35713688 PMC9203774

[B10] Cuna A. C. Reddy N. Robinson A. L. Chan S. S. (2018). Bowel ultrasound for predicting surgical management of necrotizing enterocolitis: a systematic review and meta-analysis. Pediatr. Radiol. 48, 658–666. 10.1007/s00247-017-4056-x 29260286 PMC5895673

[B11] Epelman M. Daneman A. Navarro O. M. Morag I. Moore A. M. Kim J. H.. (2007). Necrotizing enterocolitis: review of state-of-the-art imaging findings with pathologic correlation. Radiographics 27, 285–305. 10.1148/rg.272055098 17374854

[B12] Erinumberger (2017). NEC Symposium Summary. NEC Society 2017. Available online at: https://necsociety.org/2017/05/10/2017-nec-symposium-summary/ (accessed April 2017).

[B13] Faingold R. (2018). Technical aspects of abdominal ultrasound and color Doppler assessment of bowel viability in necrotizing enterocolitis. Pediatr. Radiol. 48, 617–619. 10.1007/s00247-018-4077-0 29368011

[B14] Faingold R. Daneman A. Tomlinson G. Babyn P. S. Manson D. E. Mohanta A.. (2005). Necrotizing enterocolitis: assessment of bowel viability with color Doppler US. Radiology 235, 587–594. 10.1148/radiol.2352031718 15858098

[B15] Gao H. X. Yi B. Mao B. H. Li W. Y. Bai X. Zhang Y.. (2021). Efficacy of abdominal ultrasound inspection in the diagnosis and prognosis of neonatal necrotizing enterocolitis. Clinics 76, e1816. 10.6061/clinics/2021/e1816 33787653 PMC7978842

[B16] Hwang M. Tierradentro-García L. O. Dennis R. A. Anupindi S. A. (2022). The role of ultrasound in necrotizing enterocolitis. Pediatr. Radiol. 52, 702–715. 10.1007/s00247-021-05187-5 34654968

[B17] Kotb M. Abdelatty M. Rashwan H. AbdelMeguid Y. Elrouby A. (2021). Intussusception in preterm neonates: a systematic review of a rare condition. BMC Pediatr. 21, 587. 10.1186/s12887-021-03065-5 34952564 PMC8709945

[B18] Lazow S. P Tracy S. A Staffa S. J Estroff J. A Parad R. B Castro-Aragon I. M. (2021). Abdominal ultrasound findings contribute to a multivariable predictive risk score for surgical necrotizing enterocolitis: a pilot study. Am. J. Surg. 222, 1034–1039. 10.1016/j.amjsurg.2021.04.025 33958200

[B19] Markel T. A. Underwood M. A. (2018). Preface: the NEC society. Semin. Pediatr. Surg. 27, 1–2. 10.1053/j.sempedsurg.2017.11.001 29275809

[B20] Miller L. E. Stoller J. Z. Fraga M. V. (2020). Point-of-care ultrasound in the neonatal ICU. Curr. Opin. Pediatr. 32, 216–227. 10.1097/MOP.0000000000000863 31851056

[B21] Muchantef K. Epelman M. Darge K. Kirpalani H. Laje P. Anupindi S. A. (2013). Sonographic and radiographic imaging features of the neonate with necrotizing enterocolitis: correlating findings with outcomes. Pediatr. Radiol. 43, 1444–1452. 10.1007/s00247-013-2725-y 23771727

[B22] Neu J. Walker W. A. (2011). Necrotizing enterocolitis. N. Engl. J. Med. 364, 255–264. 10.1056/NEJMra1005408 21247316 PMC3628622

[B23] Palleri E. Kaiser S. Wester T. Arnell H. Bartocci M. (2017). Complex fluid collection on abdominal ultrasound indicates need for surgery in neonates with necrotizing enterocolitis. Eur. J. Pediatr. Surg. 27, 161–165. 10.1055/s-0036-1580702 27019149

[B24] Patel R. M Kandefer S. Walsh M. C. Bell E. F. Carlo W. A. Laptook A. R.. (2015). Causes and timing of death in extremely premature infants from 2000 through 2011. N. Engl. J. Med. 372, 331–340. 10.1056/NEJMoa1403489 25607427 PMC4349362

[B25] Phad N. Trivedi A. Todd D. Lakkundi A. (2014). Intestinal strictures post-necrotising enterocolitis: clinical profile and risk factors. J. Neonatal Surg. 3, 44. 10.47338/jns.v3.134 26023515 PMC4420333

[B26] Silva C. T Daneman A Navarro O. M Moore A. M Moineddin R Gerstle J. T. (2007). Correlation of sonographic findings and outcome in necrotizing enterocolitis. Pediatr. Radiol. 37, 274–282. 10.1007/s00247-006-0393-x 17225155

[B27] Singh Y. Tissot C. Fraga M. V. Yousef N. Cortes R. G. Lopez J.. (2020). International evidence-based guidelines on Point of Care Ultrasound (POCUS) for critically ill neonates and children issued by the POCUS Working Group of the European Society of Paediatric and Neonatal Intensive Care (ESPNIC). Crit. Care 24, 65. 10.1186/s13054-020-2787-9 32093763 PMC7041196

[B28] Tam A. L. Camberos A. Applebaum H. (2002). Surgical decision making in necrotizing enterocolitis and focal intestinal perforation: predictive value of radiologic findings. J. Pediatr. Surg. 37, 1688–1691. 10.1053/jpsu.2002.36696 12483631

[B29] Taskinlar H. Gündogdu G. Celik Y. Avlan D. Nayci A. (2014). Challenging diagnosis between intussusception and necrotizing enterocolitis in premature infants. Pediatr. Int. 56, e1–3.937. 10.1111/ped.12311 24894937

[B30] Tracy S. A Lazow S. P Castro-Aragon I. M Fujii A. M Estroff J. A Parad R. B. (2020). Is abdominal sonography a useful adjunct to abdominal radiography in evaluating neonates with suspected necrotizing enterocolitis? J. Am. Coll. Surg. 230, 903–911. 10.1016/j.jamcollsurg.2020.01.027 32081753

[B31] van Druten J. Khashu M. Chan S. S Sharif S. Abdalla H. (2019). Abdominal ultrasound should become part of standard care for early diagnosis and management of necrotizing enterocolitis: a narrative review. Arch. Dis. Child. Fetal. Neonatal. Ed. 104, F551–F559. 10.1136/archdischild-2018-316263 31079066

